# Injuries related to pets, exotic animals, and falconry in Qatar

**DOI:** 10.5339/qmj.2023.27

**Published:** 2023-11-06

**Authors:** Suha Turkmen, Guillaume Alinier, Amr Mohammed Elmoheen, Alamgir Ahmed Qureshi, Benny Remi Ponappan, Mohamed Bahgat, Rashid Khan, Aftab Azad

**Affiliations:** Department of Emergency Medicine, Qatar University, Doha, Qatar. Email: drsuhaturkmen@hotmail.com ORCID ID: 0000-0002-0557-6071; Hamad Medical Corporation Ambulance Service, Doha, Qatar; Weill Cornell Medicine-Qatar, Education City, Doha, Qatar; School of Health and Social Work, University of Hertfordshire, College Lane, Hatfield, UK; Faculty of Health and Life Sciences, Coach Lane Campus, Northumbria University, Newcastle, Newcastle upon Tyne, UK; Department of Emergency Medicine, Hamad Medical Corporation, Doha, Qatar

**Keywords:** pet, exotic animal, falconry, injury

## Abstract

Introduction: Pets and exotic animals are increasingly popular all over the world. Some of these animals may cause injuries to their owners or other people during interactions. Both injuries and systemic infections always present diagnosis and treatment challenges. Emergency physicians´ clinical experience in managing patients with injuries caused by pets and exotic animals, in particular, is limited; hence, we believe that it is a domain to explore in a Middle Eastern country to help raise awareness and provide reminders as to the best evidence-based medical practice.

Methods: Hamad Medical Corporation’s hospital records of patients treated between 2015 and 2022 were analyzed retrospectively. Cases whose diagnosis was recorded as injuries caused by animals kept as pets were included in the study. Patients were evaluated in terms of demographic characteristics, type of injury, injury locations, injury severity, treatments applied, and complications. Descriptive statistics were carried out, and findings were expressed as percentages in a frequency table.

Results: Following a search of the electronic patient records during the period of interest, 43 patients were found to have sought treatment following an injury caused by a pet or domestic exotic animal. The mean age of the patients was 23.5 years; about three-quarters were male, and approximately half were children. All injuries were minor, and 74.4% were skin abrasions. The most commonly injured body parts were the hand, the leg, and facial injuries. It was observed that cats caused 53.5% of the injuries, whereas falcons were involved in 11.6% of the cases. All patients were prescribed prophylactic antibiotics, and 60.5% were administered a tetanus injection.

Conclusion: Despite our study spanning over seven years, a relatively low number of patients reported to the government hospitals´ emergency departments. The injuries are most commonly caused by cats and often involve children and animal bites to hands. The key recommendations are for parents or childminders to always supervise children when interacting with animals, be particularly cautious, and wear some form of protection when handling pets and domestic, exotic animals. Whether it is a bite or a scratch, healthcare professionals should always anticipate the potential risk of infection, treat the patient accordingly, and prescribe prophylactic antibiotics.

## Key messages

### What Is Already Known on the Study Topic?

- Bites and scratches are common injuries, often from pets such as dogs and cats. However, there is also an increasing class of injuries among people who keep exotic pets.
- Injuries caused by animals and systemic infections that may result always carry difficulties in terms of diagnosis and treatment.

### What This Study Adds?

- Injuries caused by pets are usually abrasions and superficial, but there is the potential for deep and systemic infection to develop.
- These injuries should be followed closely. It should be kept in mind that exotic animals have different microflora.

### How This Study Might Affect Research, Practice, or Policy?

- Treatment practices must be adapted to cover the risk of unusual and potentially devastating infections.
- The animal-related injuries are most commonly caused by cats and often involve children and hand bites. They are generally of a low severity level, with complications only reported in one patient in our study population. The key recommendations are for parents or childminders always to supervise children when interacting with animals, ensure that pets have the vaccinations they require, be particularly cautious, and wear some form of protection when handling pets and domestic, exotic animals as they can be unpredictable. Healthcare professionals should always pre-empt the risk of infection, consider prescribing antibiotics, check for tetanus immunization, and recommend that the patient closely monitor the injury.

## Introduction

Living with pets has many benefits for human health. For example, it helps control hypertension, improves our lipid profile, and promotes exercise.^1^ In addition, from a psychological point of view, it reduces people’s feelings of loneliness and enables them to connect to life.^2^ Nowadays, both city life and the COVID-19 pandemic have increased our interest in pets.^3^ However, the diversity of the animal species that can be owned gradually increases. Reptiles, arthropods, tropical fish, and birds, which actually belong to the wild environment as exotic animals, become increasingly popular pets.^4^ Although it may be linked to regional tradition and culture, there are risks associated with keeping wild or exotic animals as pets, as they may cause injuries to their owners or handlers.^5^ Falconry, for example, is a common practice in the Middle East and parts of Asia.^6^ In Qatar, as in other countries, there are regulations for adopted animals. It is necessary to obtain the permits required for pets, to register these animals, to carry out health checks, and to vaccinate them. However, special permits are required for exotic animals. The relevant government body in Qatar in charge of these regulations is the Ministry of Municipality and Environment’s Department of Animal Resources. Although the care of these animals has been experienced over time, both injuries and systemic infections always carry difficulties in terms of diagnosis and treatment. Bites and scratches are common injuries, often from pets such as dogs and cats. However, there is also an increasing number of injuries among people who keep exotic animals as pets. Between 2004 and 2010, the National Health Service in England alone saw 760 consultations, 709 admissions, and 2,121 hospital days due to injuries caused by exotic pets.^7^ Globally, the Middle East has become renowned as a region driving the demand for exotic pets.^8^ Despite the potential dangers they cause to their owners and although laws are in place in the region banning or regulating the ownership of some wild animals as pets, especially endangered and at-risk species, these are not very strong deterrents, and the efficacy of the laws remains questionable.^9^ From a healthcare point of view, regulations are in place in Qatar, requiring pet owners to officially register their pets and get them followed up by a veterinarian. It contributes to the animals´ well-being and helps prevent disease transmission between them and human beings.

In addition, treatment practices must be adapted to cover the risk of unusual and potentially devastating infections.

**Figure 1.**
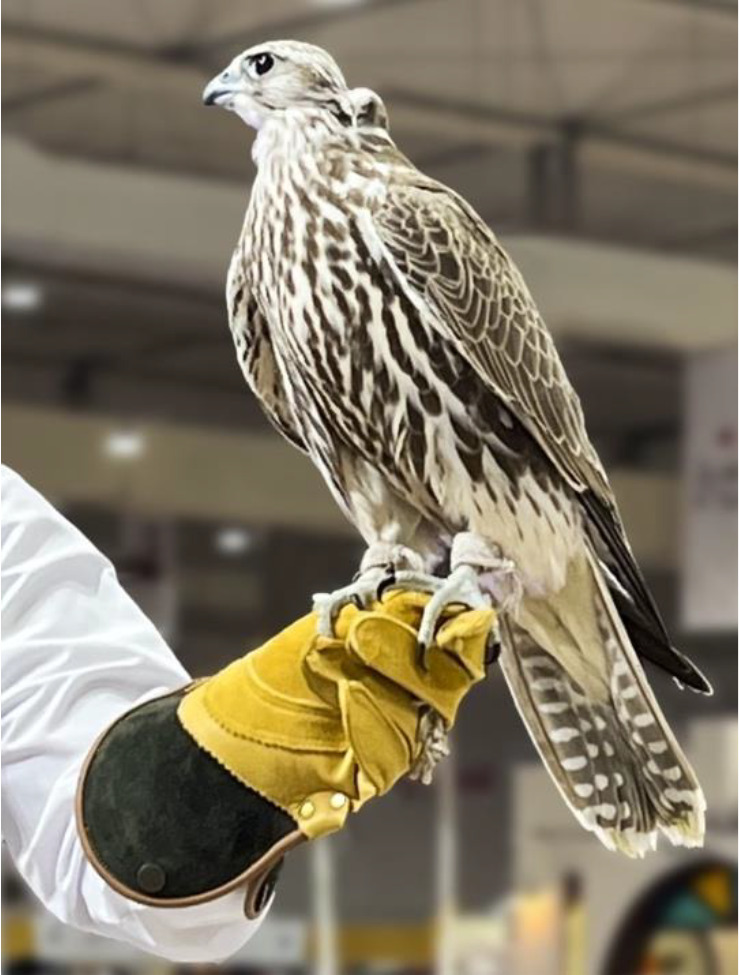
A falcon from the falconry exhibition in Qatar.

Falcons are birds of prey in the genus Falco, which includes about 40 species.^10^ Falconry hunting is hunting wild animals in their natural state and habitat by a trained bird of prey (Figure 1). It is widely found on all continents of the world except Antarctica. It has been recorded that falcons, the fastest creatures in the world, reach 400 km/h during diving.^11^ Because of their speed, they have been used for hunting since ancient times. Small animals such as birds, squirrels, and rabbits are usually hunted with falcons. Falconry is an icon of Arab culture, and Qatar is a country where falconry is part of the local culture.^6^ Historically, falconry was a popular sport and status symbol among the nobility of the Middle East. Over time, this traditional Arabian sport has grown all over Europe. In 2010, the United Nations Educational, Scientific and Cultural Organization (UNESCO) inscribed falconry as a living human heritage element of 11 countries.^12,13^

The wounds caused by exotic birds and falcons can be deep and have a potentially high infection risk. Such birds can carry a broad spectrum of zoonosis that can be transmitted to humans.^14^ As an example of this type of injury, parrot-related injuries are encountered in the literature.^15^ Contamination of parrot saliva and other wound debris through broken skin can cause serious illness if left untreated. While the principles of prompt and thorough medical evaluation, antibiotics, and possible surgical washing and debridement apply, wounds caused by exotic pets warrant further consideration. Understanding the underlying diversity of microbiological pathogens following such injuries is essential to guide optimal treatment strategies. Wound management and wound care are important aspects of these kinds of wounds.^15^ Reported injuries are rare, but there is a need to raise awareness about these types of injuries and the associated risks of infection. The aim of this retrospective study, which was conducted in the State of Qatar over seven years, is to describe the locations, types, and severity of injuries caused by pets and domestic exotic animals reported to the National Ambulance Service and the public hospitals´ emergency departments (EDs).^16,17^

## Materials and Methods

In this study, hospital records of patients treated in the ED between 2015 and 2022 were analyzed retrospectively. Data were collected from the electronic healthcare system and recorded into research forms. Ethical approval was obtained from Hamad Medical Corporation’s (HMC) Medical Research Centre (MRC-01-22-331). Cases whose diagnosis was recorded as domestic and pet injury were included in the study. Subsequent hospital records of the patients were reviewed for complications. Non-domestic arthropod, reptile, and wild animal injuries were excluded from the study, while those caused by domestic exotic animals were included. Patients were evaluated in terms of demographic characteristics, type of injury, injury locations, injury severity, treatments applied, and complications. The types of animals that caused the injury were determined. Descriptive statistics were expressed as percentages in a frequency table. Numerical data were expressed in terms of mean and standard deviation.

## Results

**Figure 2.**
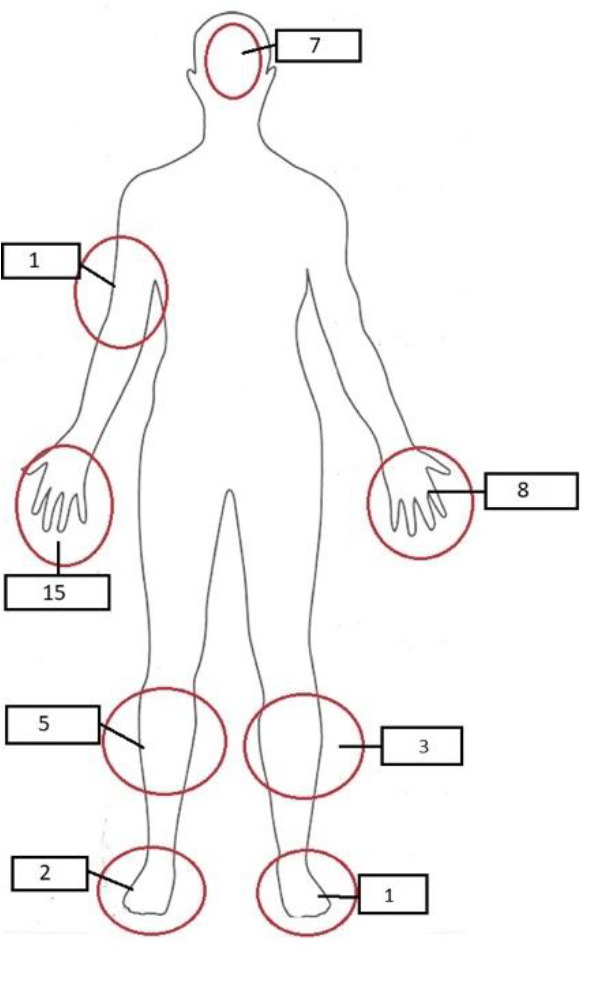
Injury locations and numbers are shown on the body diagram.

Over seven years, 43 patients were found to have sought treatment from ED following an injury caused by a pet or domestic exotic animal. The mean age of the patients was 23.5 years; approximately half of them were children, and nearly three-quarters were male. Demographic characteristics are shown in Table 1. The most commonly injured body part was the hand, followed by the leg and injuries to the face. All reported injury sites are shown in Figure 2. All injuries were in the form of superficial injuries and consisted of simple abrasions and shallow bites on the skin and consisted of superficial injuries in terms of severity. It was observed that 53.5% of the injuries were caused by cats and 16.3% by dogs, whereas falcons were involved in only 11.6% of the cases. The hospital records of the cases were reviewed after the first emergency presentation. Systemic infection, thought to be transmitted by the respiratory tract, was observed in only one case without any injury. *Chlamydia pneumonia* was considered in this patient, who was a Falcon caregiver. It was observed that the patient developed massive pleural effusion. The patient was discharged without any further complications.

## Discussion

Although our retrospective study extended over seven years with data from the public hospitals that provide most emergency healthcare services across Qatar, we found only 43 patients who had sustained injuries caused by a pet or domestic exotic animal requiring an ED visit (Table 1). Even if 50% of people who are the victims of injuries caused by animals seek treatment from primary public care or private healthcare facilities, this can be considered a low incidence rate considering a total average population of around 2.5 million inhabitants. This can be partially explained by the fact that a significant proportion of Qatar’s population comprises blue-collar expatriate male workers who often live in shared accommodations or other forms of housing where pets are not allowed.^18^ Although we do not have any data regarding the number of pets or domestic, exotic animals in Qatar or any estimate of the number of households with animals, cats have previously been reported as being the most commonly kept pets, which coincides with our findings showing that cats had caused 53.5% of the reported injuries.^19^ According to our data, children and young adults are the most frequently exposed to pet-related injuries. For this reason, it should be emphasized that children should have contact with these animals under adult supervision and with pet owners, as was recommended in other studies.^20^ Unsurprisingly, the hand is the most frequently injured body part (55.8%) as it is the limb we extend to touch or carry a domestic animal (Figure 1). Although no further issues developed following these hand injuries, the possibility of developing complications is quite high.^21^ For this reason, more care should be taken when people are in contact with pets, especially domestic exotic animals. Protections such as gloves should be used when handling animals, which can be unpredictable. In general, pet owners need to be educated about transmittable diseases and how this can be better controlled through animal vaccination and proper hygiene practices.^19^ While most pet-related injuries are minor abrasions or bites, as per the findings from our study, serious injuries have also been reported in the literature.^22^ The force of a bite from a strong or large animal may cause a fracture or crush injury, especially to the hands. Secondary to the injury are the risks of compartment syndrome or deep soft tissue infection from bacteria or pathogens that may be transmitted by animals or due to the environment, some of which could cause significant necrosis and even gangrene.^22^

It should not be forgotten that exotic birds, such as parrots and hawks, can cause serious injuries. A case report reported an open finger fracture due to a parrot bite. Although open fractures have the potential for serious infection, no complications occurred in this case.^23^ The parrot can exert approximately 200 pounds per square inch of pressure with its bite, which can cause crush injury. In another case report, a case of compartment syndrome developed due to a parrot bite without any fracture and in a very short period.^24^ In another case report, a hawk caused a puncture injury and superficial abrasions on someone’s hand who was admitted to the hospital two days after the event. The patient developed a severe soft tissue infection, and surgery was recommended, considering that he had progressed to compartment syndrome. However, because the patient refused surgery, he was hospitalized and treated with parenteral broad-spectrum antibiotics for three days. The infection regressed, and he was eventually treated with oral antibiotics.^15^ In another case report, a 5-cm nodule developed and was excised from a patient whom a cockatoo parrot had bitten. The culture results of the nodule revealed *Mycobacterium chelonae*. Despite the sensitive antibiotic regimen, nodules developed in the patient’s forearm. These lesions were also excised, and the patient could be treated after 12 months of antibiotic therapy.^26^

Wound care is similar for all animal bites or scratch injuries, and the wound should be cleaned appropriately and then closely monitored for infection. Deep injuries require surgical debridement and washout.^22^ Prophylactic antibiotics should be started for all injuries with a risk of infection. This prophylaxis can be achieved with 3 or 5 days of amoxicillin-clavulanate (875/125 mg twice daily). In case of penicillin allergy, doxycycline (100 mg, twice daily) plus metronidazole (500 mg, three times daily) can be used. Pastorella and anaerobic pathogens should be considered in exotic animal injuries. Prophylaxis can be provided with moxifloxacin (400 mg daily) as well.^23^

Another critical situation is zoonotic systemic infections. Notably, 61% of human diseases are considered zoonotic origin, and 75% of globally occurring human diseases are associated with wild animals. In systemic infections or zoonotic infections transmitted by direct, indirect, or aerosol means, symptoms usually appear with flu-like symptoms, and many cases cannot be diagnosed. In all systemic infections, patients should be questioned in terms of animal contact, and potential pathogens should be considered. For example, many *Salmonella* infections are pet turtle-related salmonellosis, and the case rates have decreased significantly when adequate precautions are taken, such as hygiene of the animals, hand hygiene, and supervising the kids.^26^ Reptile-related salmonellosis has been shown as another cause of salmonella infection. *Escherichia coli* gastroenteritis, campylobacteriosis, vibriosis, giardiasis, and cryptosporidiosis can be seen as other systemic zoonotic infections that reptiles, fish, and birds can transmit. However, another essential condition is serious allergic reactions, which are life-threatening and result from bites and stings, especially caused by reptiles, arthropods, and exotic fish.^26^ One of the zoonotic infections related to exotic birds is psittacosis. Although psittacosis is a rare disease that parrots transmit, the actual number of cases is believed to be higher than reported because it is a complex diagnosis. Psittacosis, also known as chlamydiosis, parrot fever, and ornithosis, is caused by *Chlamydophila psittaci*, with an incidence of 40% in all birds.^27^ Contamination in parrots’ respiratory and gastrointestinal tracts is most common through bites. People presenting with atypical pneumonia with systemic malaise and long-term sequelae include myocarditis and meningoencephalitis. Tetracyclines are the mainstay of therapy for parrots and humans, but chloramphenicol and erythromycin are valid alternatives where tetracyclines are contraindicated.^27^ Our case involved a falcon caregiver who developed a massive pleural effusion. Although the diagnosis could not be revealed by chlamydia antigen test, it was thought to be *C. pneumonia*. He was hospitalized, treated with parenteral antibiotics, and discharged after the clinical picture improved.

Pasteurella, Mycobacterium, Cryptococcus, erysipeloid, and Newcastle disease can be counted as other causes of systemic infection. *Pasteurella multocida*, isolated from the nasopharynx of parrots, causes pasteurellosis and can be transmitted through bites or respiratory droplets. If left untreated, it can cause systemic discomfort, and antibiotic treatment options include amoxicillin/clavulanic acid, doxycycline, amoxicillin, or quinolones. Nontuberculous mycobacteriosis caused by *Mycobacterium avium* is transmitted through beaks and claws. Most infections are asymptomatic, but treatment includes macrolides, ethambutol, and rifabutin.^22^ Other zoonoses, including Cryptococcus, erysipeloid, and Newcastle disease, are found in soil contaminated with infected feces. In the presence of broken skin, they can cause localized and systemic infections. Erysipeloid treatment is with penicillin or erythromycin; Fluconazole is the first choice for Cryptococcus.^25^ It is thought that the parrots come from the same root as the falcon family, and similar clinical pictures may also be present in the falcon family. Hygiene, avoiding close contact, and protective measures are essential to prevent systemic infections.^28^

Tetanus prophylaxis is required in cases where all skin integrity is impaired. However, there is always the potential for rabies in injuries caused by animals in contact with the wild environment, as bites, scratches, abrasions, or contact with animal saliva via mucous membranes can transmit rabies.^29^ In fact, rabies appears to be a condition that causes infection in warm-blooded mammals. However, it has also been shown that birds are infected with rabies *in vitro*.^30^ Upon discharge of patients with a course of prophylaxis antibiotics, they should be advised to monitor the healing of their injury and return to the ED should they start developing fever or if there is persistent soreness, swelling, and redness.

Our study has limitations. Injuries related to pets or domestic, exotic animals appear relatively rare in Qatar due to the population demographics, including a substantial proportion of migrant workers who cannot keep pets in their accommodation. Our study used secondary data recorded across HMC facilities and hence does not include cases treated by private clinics and hospitals or the Primary Healthcare Corporation, a common point of call for minor injuries.^31^ It has also been noted in another study that limited or poor documentation makes the scale of the problem difficult to evaluate.^32^

## Conclusion

Despite our study spanning over seven years, a relatively low number of patients reported to the government hospitals´ EDs in Qatar after having sustained an injury caused by a pet or domestic exotic animal. The injuries of the cases included in our study were minor skin abrasions and superficial injuries, mainly to the hands. They were often caused by cats and involved children and young adults. The injuries were generally of a low severity level, with complications reported in only one patient. The key recommendations are for parents or childminders always to supervise children when interacting with animals, ensure that pets have the vaccinations they require, be particularly cautious, and wear some form of protection when handling pets or domestic, exotic animals. Whether it is a bite or a scratch, healthcare professionals should always anticipate the potential risk of infection and treat the patient accordingly by prescribing antibiotics and advising them to seek further medical assistance if the injury location does not heal rapidly.

## Author contributions

Suha Turkmen and Guillaume Henri Jean Alinier: study concept and design. Suha Turkmen, Alamgir Ahmed Qureshi, and Benny Remi Ponappan: data acquisition. Guillaume Henri Jean Alinier, Amr Mohammed Elmoheen, and Aftab Azad: data analysis. Suha Turkmen, Guillaume Henri Jean Alinier, Rashid Khan, Kamal Majed, and Mohamed Bahgat: drafting and critical revision of the manuscript. All authors approved the final manuscript.

## Conflict of interest statement

The authors declare that there are no competing interests.

## Consent to participate

All authors will sign the consent.

## Ethical approval

Ethical approval was obtained from the Hamad Medical Corporation’s (HMC) Institutional Review Board (MRC-01-22-331).

## Figures and Tables

**Table 1.** Demographic data.

**Demographic data**	**Number (*N* = 43)**	**Percentage**
Gender		
Male (MA 26.13 – SD 14.46)*	32	74.40%
Female (MA 18.55 – SD 14.92)*	11	25.60%
Age groups (years)		
Pediatric (0–17)	19	44.20%
Adult (18–64)	23	53.50%
Elderly >65	1	2.30%
Animal type		
Cat	23	53.50%
Dog	7	16.30%
Falcon	5	11.60%
Rabbit	3	7.00%
Camel	2	4.70%
Fox	1	2.30%
Monkey	1	2.30%
Rat	1	2.30%
Body side		
Right	23	53.50%
Left	15	34.90%
Midline	4	9.30%
Not applicable	1	2.30%
Body part		
Hand	24	55.80%
Leg	8	18.60%
Face	7	16.30%
Foot	3	7.00%
Not applicable	1	2.30%
Type of injury		
Skin abrasion or superficial skin damage	32	74.40%
Bite-related puncture wound	10	23.20%
Systemic infection (no injury)	1	2.30%
Severity level		
Minor	43	100%
Moderate	0	0%
Major	0	0%
Severe	0	0%
Treatment provided		
Wash out	15	34.90%
Antibiotic therapy	43	100%
Tetanus prophylaxis	26	60.50%
Suturing	0	0%
Orthopedic intervention	0	0%
Complication		
None	42	97.70%
*Chlamydia pneumonia*	1	2.30%

* Male and female mean age (MA) in years and standard deviation (SD) are shown.
